# Kif2a regulates spindle organization and cell cycle progression in meiotic oocytes

**DOI:** 10.1038/srep38574

**Published:** 2016-12-19

**Authors:** Zi-Yun Yi, Xue-Shan Ma, Qiu-Xia Liang, Teng Zhang, Zhao-Yang Xu, Tie-Gang Meng, Ying-Chun Ouyang, Yi Hou, Heide Schatten, Qing-Yuan Sun, Song Quan

**Affiliations:** 1 Center for Reproductive Medicine; Department of Ob/Gy; Nanfang Hospital; Southern Medical University, Guangzhou City, 510515 China; 2 State Key Laboratory of Stem Cells and Reproductive Biology, Institute of Zoology, Chinese Academy of Sciences, Beijing, 100101 China; 3 Department of Veterinary Pathobiology, University of Missouri, Columbia, MO 65211, USA

## Abstract

Kif2a is a member of the Kinesin-13 microtubule depolymerases. Here, we report the expression, subcellular localization and functions of Kif2a during mouse oocyte meiotic maturation. Immunoblotting analysis showed that Kif2a was gradually increased form GV to the M I stages, and then decreased slightly at the M II stage. Confocal microscopy identified that Kif2a localized to the meiotic spindle, especially concentrated at the spindle poles and inner centromeres in metaphase and translocated to the midbody at telophase. Kif2a depletion by siRNA microinjection generated severely defective spindles and misaligned chromosomes, reduced microtubule depolymerization, which led to significant pro-M I/M Iarrest and failure of first polar body (PB1) extrusion. Kif2a-depleted oocytes were also defective in spindle pole localization of γ-tubulin and showed spindle assembly checkpoint (SAC) protein Bub3 at the kinetochores even after 10 hr extended culture. These results demonstrate that Kif2a may act as a microtubule depolymerase, regulating microtubule dynamics, spindle assembly and chromosome congression, and thus cell cycle progression during mouse oocyte meiotic maturation.

Mouse oocyte maturation is a complex and precisely orchestrated process^1^,^2^. Germinal vesicle breakdown (GVBD) represents the initiation of oocyte maturation. After GVBD, microtubules assemble around chromosomes into a fusiform bipolar spindle at the MI (metaphase of the first meiotic division) stage, followed by spindle migration to the cortex, first polar body extrusion and metaphase II (metaphase of the second meiotic division) spindle formation^3^. Prophase I arrest release and progression through M I are two critical stages for mammalian oocyte maturation^3^.

The bipolar spindle is a microtubule-based apparatus that is responsible for accurate chromosome congression and segregation in mitosis and meiosis^4^. The formation of meiotic spindles relies on concerted actions of multiple microtubule organizing centers (MTOCs), microtubule depolymerases such as the kinesin-13 family of proteins, and of microtubule-associated proteins (MAPs)^5^,^6^. MTOCs contain hundreds of proteins, including γ-tubulin, pericentrin, GM130, p38α and numerous others^7^,^8^. In the mouse oocyte, MTOCs are dynamically sorted and distributed along the spindle before clustering toward the two poles during M I to intermediates before elongation and establishment of the barrel-shaped acentriolar meiotic spindle^9^,^10^.

The dynamic polymerization and depolymerization of microtubules is crucial for spindle assembly, chromosome congression and segregation^11^. In anaphase, as the kinetochores bound microtubules shorten through the loss of tubulin subunits from plus and minus ends of the spindle, chromosomes separate and move poleward through their attachment sites at kinetochores^12^. The kinesin-13 family of MT depolymerases (Kinl kinesins), defined as the conserved motor domain positioned in the middle of the protein, lacks motility and stimulate microtubule depolymerization by inducing conformation changes in protofilament structure at both plus and minus ends of microtubules^13^,^14^,^15^. Mammals have three members of the kinesin-13 family, including Kif2a, Kif2b, and MCAK/Kif2c. These proteins localize to different components of the spindle, and their concerted action controls many processes, such as bipolar spindle organization, kinetochore-microtubule capture, and chromosome movement in mitosis^16^. There is growing evidence that the kinesin-13 family plays important roles in both transformation and resistance to spindle poison chemotherapeutics, especially the derivatives of paclitaxel^15^.

Kif2a is localized to spindle microtubules, concentrates at spindle poles in Hela cells^11^ and at kinetochores in Xenopus cells^17^. Kif2a is found abundantly expressed in neurons, in which Kif2a depolymerizes MTs to suppress the growth of axonal collateral branches^18^,^19^. When Kif2a^−^/^−^ mice are born, they show brain abnormalities, and human retardation syndrome maps to the Kif2a locus^18^,^20^. It is also a critical regulator of mitotic spindle dynamics. Kif2a is also required for spindle assembly in mitosis where it is thought to depolymerize microtubules at the minus ends at the poles, thereby maintaining spindle length^6^,^21^. Depletion of Kif2a generates monopolar spindles in Hela cells^11^, while it contributes to multipolar spindles and failure of pole coalescence in Xenopus embryos^17^. The pesticide dichlorvos induced spindle monopolarity by inhibiting the depolymerizing activity of Kif2a at centrosomes^22^.

Kif2a activity is regulated positively by Plk1 and negatively by Aurora A through phosphorylation which is also regulated through an interaction with the inner centromere Kin-I stimulator (ICIS)^23^,^24^. Aurora B activity gradient determines the spatial distribution of Kif2a and inhibits Kif2a-mediated depolymerization in a complex manner in the Hela KyoTo line to ensure faithful cell division^25^. Importin α regulates the concentration of Kif2a in Xenopus embryogenesis to help mitotic spindle adaptation to changing cell dimensions during early development in Xenopus embryogenesis^26^.

While it is known that Kif2a plays important roles in spindle integrity, proper chromosome segregation, pole coalescence in mitosis, as of now, whether Kif2a participates in meiotic spindle assembly, subsequent chromosome alignment and segregation remains unkown. In this study, we investigated the expression, localization and potential roles of Kif2a in mouse oocyte meiosis, using siRNAs to knockdown the expression of Kif2a. We found that the expression level of Kif2a was gradually increased form GV to the M I stages, and then decreased slightly at the M II stage and localized to both spindle microtubules and centromeres, concentrated at spindle poles, and was required for spindle assembly, spindle pole formation, chromosome segregation and the first polar body extrusion during mouse oocyte meiotic maturation.

## Results

### Expression and localization of Kif2a during mouse oocyte meiotic maturation

**Figure 1:**
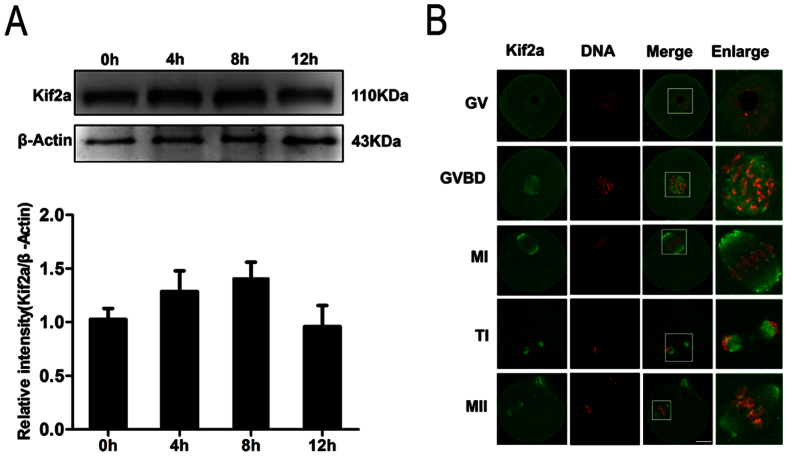
Expression and subcellular localization of Kif2a during mouse oocyte meiotic maturation. (**A**) Western blotting results for expression of Kif2a were cropped gels. Oocytes were collected after culture for 0, 4, 8, 12 h, corresponding to the GV, Pro-M I, M I and M II stages, respectively. The molecular weight of Kif2a and β-actin were 110 kDa and 43 kDa, respectively. Each sample contained 200 oocytes. Full-length gels are presented in Supplementary Figure 1. The intensity of Kif2a/β-actin was assessed by grey level analysis. (**B**) Subcellular localization of Kif2a shown by immunofluorescent staining and confocal microscopy. Oocytes at various stages (GV, GVBD, M I,T Iand M II) were stained with antibody against Kif2a. Green, Kif2a; red, DNA; Magnifications of the boxed regions are shown. Bar = 20 μm.

To investigate the expression of Kif2a during mouse oocyte meiotic progression, we cultured mouse oocytes for 0, 4, 8, 12 h, corresponding to the GV, Pro- M I, M I and M II stages, respectively. As shown in Fig. 1A, the expression level of Kif2a was low at GV, and gradually increased form GV to the MI stages, and then decreased slightly at the M II stage.

Next, we investigated the subcellular location of Kif2a during meiotic maturation by immunofluorescent staining. As shown in Fig. 1B, at the GV stage, Kif2a was mainly distributed in the nucleus. After GVBD, Kif2a began to accumulate in the vicinity of the condensed chromosomes, displaying also faint staining around centromeres of chromosomes, but the signal was just slightly above the background level. At the M I stage, when the chromosomes were aligned at the equatorial plate, Kif2a localized to spindle microtubules, being concentrated at poles, but also accumulated strongly around centromeres of chromosomes. Kif2a was stained at the midbody at telophase I. At the M II stage, Kif2a was again localized at the spindle poles and the spindle microtubules, and also around the centromeres. This localization pattern for Kif2a is slightly different from that reported for Kif2a in mitosis^11^,^17^.

**Figure 2:**
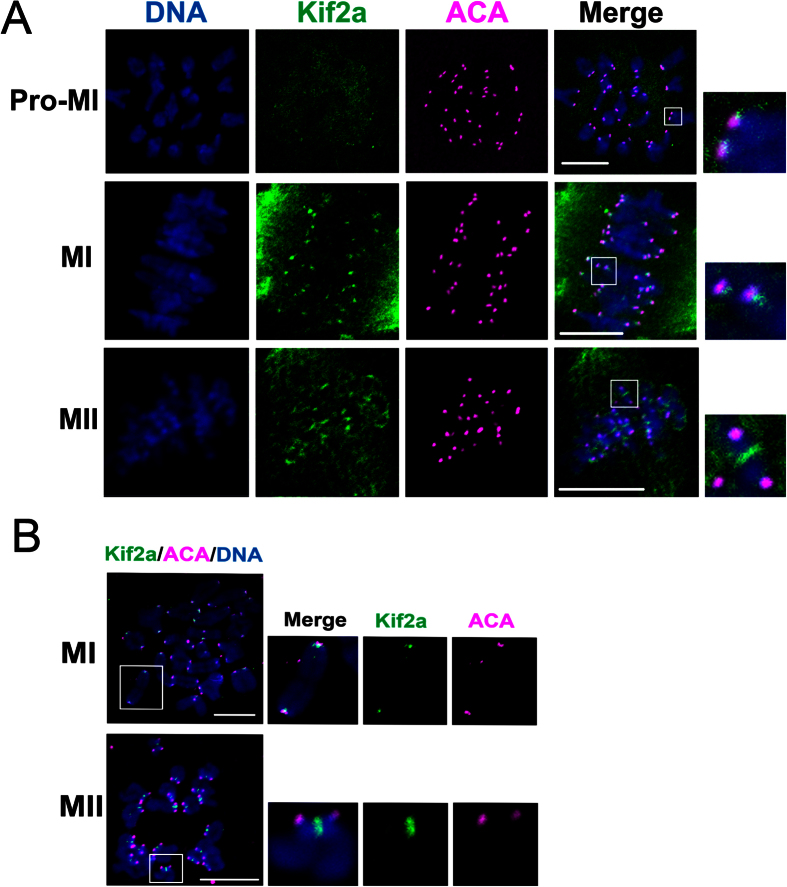
Localization of Kif2a on chromosomes. (**A**) Co-localization of Kif2a with ACA at Pro-M I,M I, and M II stages. Oocytes cultured to 4 h (Pro-M I), 8 h (M I), and 12 h (M II) were stained for Kif2a (green), ACA (pink), and DNA (blue). Bar = 10 μm. (**B**) Colocalization of Kif2a and ACA at M I and M II stages. Oocytes were cultured to M I and M II stages, then chromosomes were spread and stained with Kif2a (green), ACA (pink), and DNA (blue). Magnifications of the boxed regions were displayed on the right of the main panel. Bar = 10 μm.

To further investigate the centromeric localization of Kif2a, oocytes were cultured to different stages, fixed and double stained with ACA (anti-centromere antibody) and anti-Kif2a antibodies. As shown in Fig. 2A, at the Pro-M I stage, Kif2a was detected faintly adjacent to the ACA signals. Strikingly, Kif2a accumulated brightly in the vicinity of ACA at the M I stage (Fig. 2A). At the metaphase II stage, Kif2a was localized at the inner centromere area between the two ACA signals of the sister chromatids (Fig. 2A).

To obtain more detailed information of Kif2a localization on chromosomes, we employed chromosome spreading and immunofluorescent staining. As shown in Fig. 2B, at the M I stage, the signal of Kif2a was detected at the inner centromere, accumulating in the vicinity of ACA, and faint staining was detected along the interchromatid axes. At the M II stage, clear Kif2a was observed at the inner centromere area between the two ACA signals of the sister chromatids. Faint Kif2a signals were detected along the chromatid arms. It further confirmed the localization of Kif2a at the inner centromeres in meiosis.

### Localization of Kif2a in mouse oocytes treated with taxol or nocodazole

**Figure 3:**
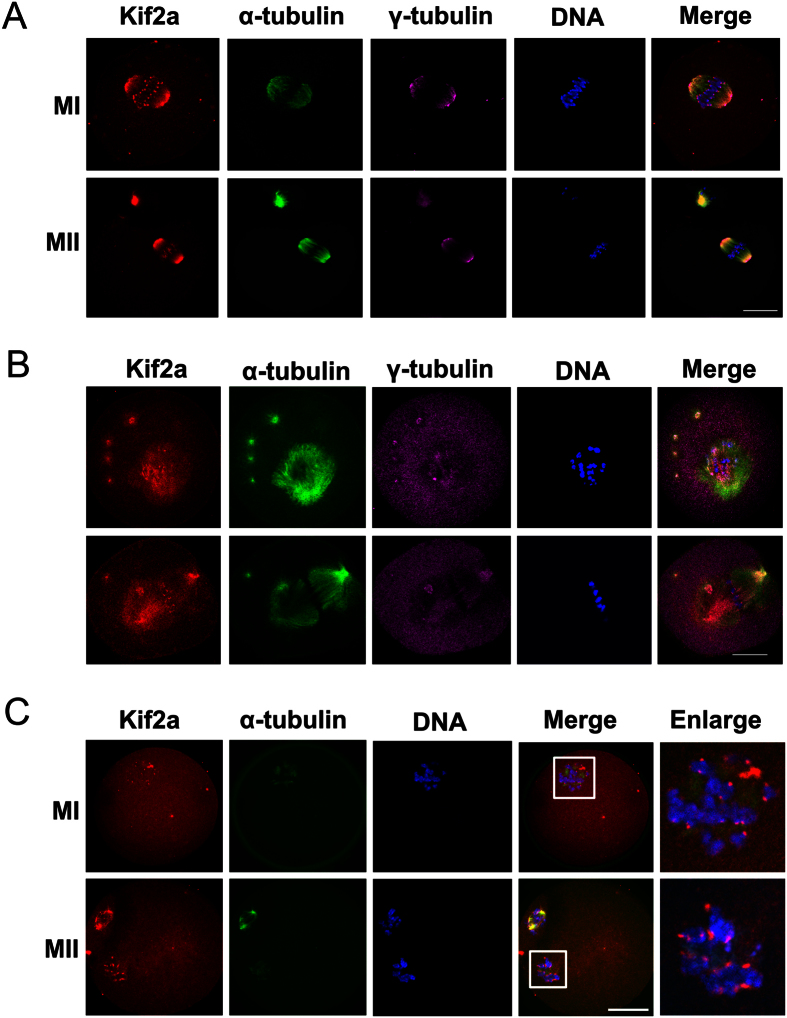
Localization of Kif2a in mouse oocytes treated with spindle perturbing agents, taxol or nocodazole. (**A**) Oocytes were collected at 8 h or 12 h of culture in M2 medium containing DMSO stock. And then triple stained with antibodies against Kif2a, γ-tubulin, and anti-α-tubulin-FITC. (**B**) Oocytes at the M I stage were incubated in M2 medium containing 10 μm taxol for 45 min and then triple stained with antibodies against Kif2a, γ-tubulin, and anti-α-tubulin-FITC. Kif2a was localized at the enlarged spindle, congressed spindle poles, centromeres, and cytoplasmic asters. (**C**) Oocytes at M I and M II stages were incubated with 20 μg/ml nocodazole in M2 medium for 15 min and then double stained for Kif2a and α-tubulin. Red, Kif2a; green, α-tubulin; pink, γ-tubulin; blue, chromatin; Magnifications of the boxed regions were displayed on the right of the main panel. Bar = 20 μm.

After determining that Kif2a was localized at spindle microtubules, with strong accumulation at the spindle poles in the M I and M II stages, we further analyzed the correlation between Kif2a localization and microtubule dynamics by employing taxol and nocodazole. As shown in Fig. 3A, in the control group, both Kif2a and γ-tubulin signals overlapped at the spindle poles, Kif2a also distributed on spindle microtubules and accumulated strongly around centromeres of chromosomes. As presented in Fig. 3B, in taxol-treated oocytes, the microtubule fibers were excessively polymerized, leading to notably enlarged spindles and multiple asters in the cytoplasm. After being treated with taxol, Kif2a was co-localized with α-tubulin and accumulated at abnormal spindle poles as well as in the cytoplasmic asters, but it also was localized at the centromeres. Kif2a and γ-tubulin signals partially overlapped at the abnormal spindle poles as well as in the cytoplasmic asters. Microtubule fibers in nocodazole-treated oocytes were entirely disassembled, and the localization of Kif2a was changed; it partly dispersed into the cytoplasm and reappeared at the inner centromere (Fig. 3C).

### Knockdown of Kif2a results in abnormal spindles and misaligned chromosomes

**Figure 4:**
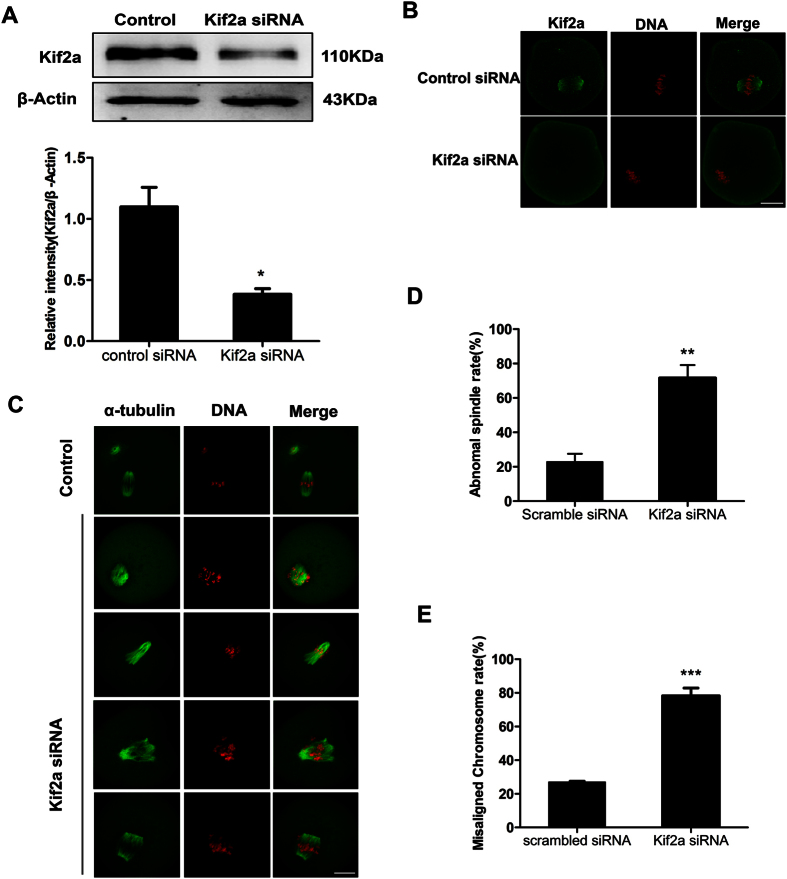
Depletion of Kif2a causes abnormal spindles and misaligned chromosomes in mouse oocytes. (**A**) Western blotting results for Kif2a after siRNA injection were cropped gels. After microinjection of Kif2a or control siRNA, oocytes were incubated in M2 medium containing 100 μm IBMX for 24 h, then washed 5 times and placed in IBMX-free M2 medium for 8 h, followed by Western blotting. Relative expression of Kif2a after siRNA injection. The intensity of Kif2a/β-actin was assessed by gray level analysis. Data are presented as Means ± SEM of 3 independent experiments (*P < 0.05). Full-length gels are presented in Supplementary Figure 2. (**B**) Confocal images showing knockdown of Kif2a in the siRNA-injection group after 24 h inhibition in 100 μm IBMX and 8 h of culture in M2 medium. A total of 53 oocytes were assessed in the Kif2a siRNA-group and 56 oocytes were assessed in the control siRNA-group. Green, Kif2a; red, chromatin. Bar = 20 μm. (**C**) Oocytes microinjected with Kif2a or control siRNA were collected at 12 h in IBMX-free M2 medium. Kif2a -depleted oocytes exhibited various kinds of deformed spindles and chromosome misalignment. Green, α-tubulin; red, chromatin; Bar = 20 μm. (**D**) The rate of oocytes with abnormal spindles in the Kif2a siRNA-injection group and control siRNA-injection group. Data are presented as Means ± SEM of 3 independent experiments (**P < 0.01). (**E**) Percentage of oocytes with chromosome misalignment in the Kif2a knockdown group and in the control group. Data are presented as mean ± SEM of 3 independent experiments (***P < 0.001).

To study the function of Kif2a, we employed specific siRNA injection to deplete Kif2a in oocytes. Western blot demonstrated that the expression level of Kif2a was notably reduced (Fig. 4A). As shown in Fig. 4B, Kif2a signal was undetectable in siRNA-injected oocytes, while it was obviously observed at the spindle in the control siRNA-injected oocytes, which is consistent with the results of the immunoblot analysis. All of the above confirmed the successful knockdown of Kif2a. In the Kif2a knockdown group, there were severe defects in spindle formation and chromosome alignment.

The aberrant spindle organization mainly included broadened spindle and abnormal spindle poles, including spindles with no poles, monopoles, and multipoles (Fig. 4C). Among the aberrant spindles, the broadened spindles accounted for 9.8%, the multipole spindle configurations accounted for 21.37%, and the major spindle defects were none- and mono- polar spindles (68.83%).

As shown in Fig. 4D, the rate of abnormal spindle formation in the Kif2a-siRNA injected group (71.63 ± 7.54%, n = 131) was significantly higher than that of the control-siRNA injected group (22.98 ± 4.5%, n = 122). Significant absence of chromosome alignment was also detected in Kif2a-depleted oocytes, including lagging chromosomes and irregularly scattered chromosomes (Fig. 4C). The incidence of the misaligned chromosomes in Kif2a siRNA-injection group (78.48 ± 7.88%, n = 131) was considerably higher than that in the control group (27.00 ± 1.0%, n = 122).

### Depletion of Kif2a causes dissociation of γ-tubulin from spindle poles

**Figure 5:**
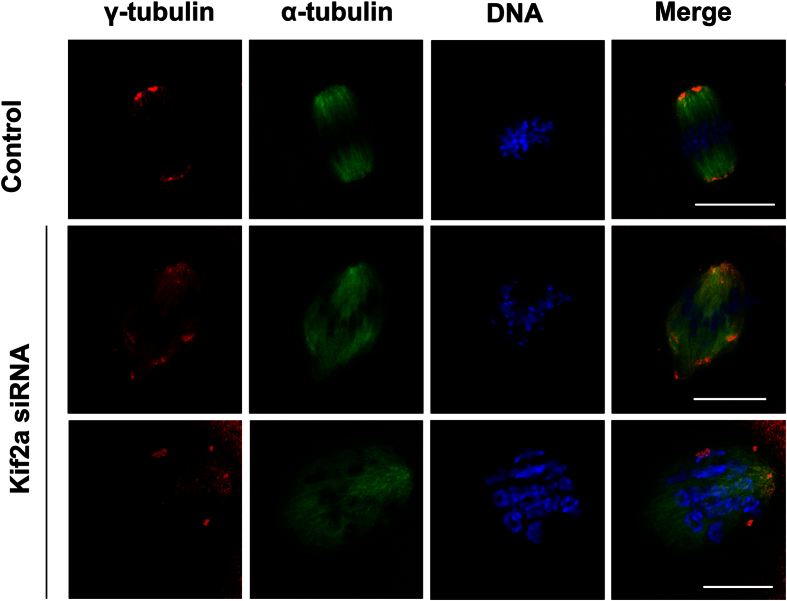
Dissociation of γ-tubulin from spindle poles in Kif2a-knockdwon oocytes. Oocytes microinjected with Kif2a or control siRNA were arrested at the GV stage in IBMX for 24 h, and incubated in IBMX-free M2 medium for 8 h, followed by double staining for α-tubulin, γ-tubulin. In the control siRNA-injected group, γ-tubulin was localized to the spindle poles at the MI stage, while in the Kif2a siRNA-injected group, γ-tubulin was dissociated form abnormal spindle poles and dispersed into the cytoplasm. Red, γ-tubulin; green,α-tubulin; blue, DNA. Bar = 20 μm.

It is well known that γ-tubulin is the MTOC-specific protein that is important for microtubule nucleation and spindle formation during mouse oocyte meiosis. We further explored the effect of Kif2a knockdown on γ-tubulin localization. As shown in Fig. 5, γ-tubulin was localized to spindle poles in the control group at the M I stage. Strikingly, after Kif2a siRNA injection, γ-tubulin was no longer accumulated at spindle poles, being irregularly distributed into the cytoplasm.

### Knockdown of Kif2a causes Pro-MI/MI arrest and decreased PB1 extrusion

**Figure 6:**
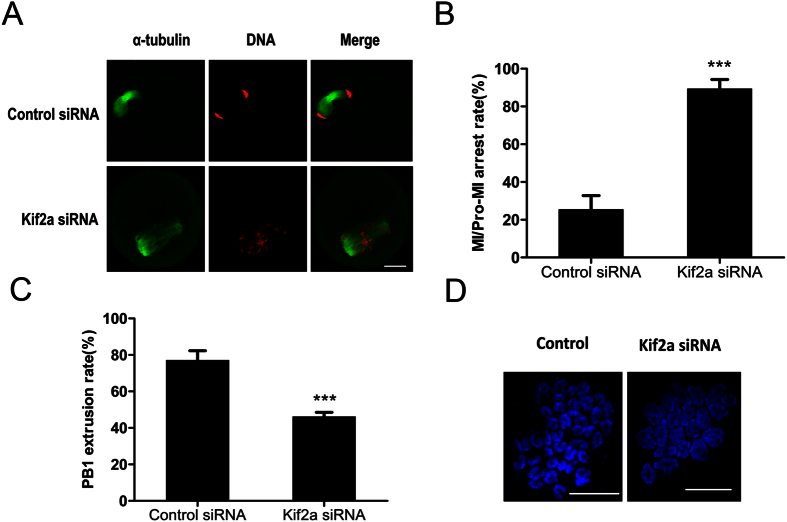
Depletion of Kif2a arrests oocytes at the Pro-MI/MI stage and decreases PB1 extrusion. Oocytes injected with Kif2a siRNA or control siRNA were cultured in fresh M2 medium for 10 h or 12 h, after being incubated in M2 medium containing 100 μm IBMX for 24 h. (**A**) Oocytes cultured for 10 h after IBMX washout in the Kif2a-depletion group were arrested at the Pro-MI/MI stage, whereas oocytes in the control group reached the AI stage. Injected oocytes were stained with α-tubulin (green); DNA (red). Bar = 20 μm. (**B**) The rate of Pro-MI/MI cultured oocytes was recorded in the Kif2a knockdown group and the control group at 10 h of culture. Data are presented as Mean ± SEM of 3 independent experiments (***P < 0.001). (**C**) Percentages of PB1 extrusion of oocytes cultured for 12 h in the Kif2a knockdown group and control group. Data are presented as Mean ± SEM of 3 independent experiments (***P < 0.001). (**D**) Chromosome spreading showed failure of homologous chromosome segregation in the Kif2a knockdown group after culture for 12 h. The percentage for bivalents was 35/46 in the knockdown group and 16/56 in the control group. Bar = 20 μm.

After microinjection of Kif2a siRNA or control siRNA, oocytes were maintained arrested at the GV stage in M2 medium containing 100 uM IBMX for 24 h; then the oocytes were continuously cultured in IBMX-free M2 medium for 10 h or 12 h. As shown in Fig. 6A, the majority of oocytes were arrested at the Pro-M I/M Istage in the Kif2a-knockdown group by 10 h, while the oocytes in the control group reached the anaphase I stage. As shown in Fig. 6B, the Pro-M I/M I block rate in the Kif2a knockdown group (89.29 ± 5.027%, n = 116) was considerably higher than that in the control group (25.35 ± 7.46%, n = 110). The PB1 extrusion in Kif2a knockdown group (46.28 ± 2.22%, n = 270) was significantly lower than that in the control group (77.25 ± 5.073%, n = 313) by 12 h (Fig. 5C).

Since the majority of oocytes arrested at the Pro-M I/M I stage in the Kif2a knockdown group, with aberrant spindles and misaligned chromosomes, we employed chromosome spreading to confirm whether the chromosomes could segregate correctly. Oocytes in both the Kif2a knockdown group and the control group were cultured for 12 h. Our results showed that chromosomes were still bivalents in the Kif2a knockdown group (10/10); in contrast, univalent chromosomes were observed in the control group (9/9), indicating completion of first meiosis (Fig. 6D).

### Knockdown of Kif2a activates SAC to prevent chromosome segregation

**Figure 7:**
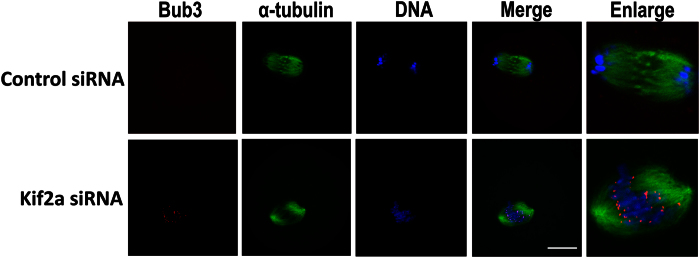
Kif2a knockdown causes activation of SAC. Oocytes from the Kif2a knockdown group and the control group were cultured in IBMX-free M2 medium for 10 h, then stained with bub3 and α-tubulin. Bub3 localization at the kinetochores of Kif2a knockdown oocytes. Red, Bub3; green, α-tubulin; blue, DNA. Bar = 20 μm.

To further explore the cause for the Pro-M I/M I arrest we analyzed the localization of Bub3, an important component of SAC. Specific signal of Bub3 was detected on chromosome kinetochores in the Kif2a-knockdown oocytes at 10 h after IBMX washout, while the control oocytes entered anaphase without signals of Bub3 on kinetochores. Detection of Bub3 signal indicated that the spindle assembly checkpoint was activated in the Kif2a-depleted oocytes (Fig. 7A).

### Kif2a destabilizes the spindle microtubules in meiosis

**Figure 8:**
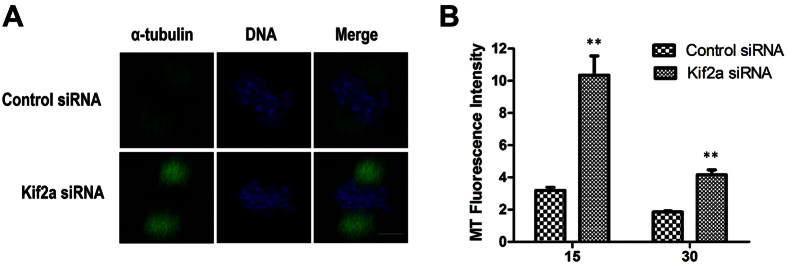
Kif2a controls spindle dynamics in mouse oocytes. Oocytes in MI stages were incubated at 4 °C for 15 min or 30 min to selectively depolymerize non-kinetochore microtubules and fixed, then stained for α-tubulin and DNA. (**A**) Single projection of z sections spanning the entire spindle width of the representative control group or Kif2a knockdown group are shown. Images for α-tubulin were obtained under a constant exposure parameter; green, α-tubulin; blue, DNA. Bar = 20 μm. (**B**) The fluorescence intensity of α-tubulin on spindles of the Kif2a knockdown group at the 15 min and 30 min time points were quantified and normalized to their respective control groups. (n = 10 cells for each quantification). (**P < 0.01).

To further understand how Kif2a is involved in spindle organization and chromosome alignment, we investigated the role of Kif2a in meiotic spindle dynamics by cold treatment. Preferential depolymerization of non-kinetochore microtubules by cold treatment confirmed that microtubule fibers remained attached to kinetochores in Kif2a-depleted oocytes. Whereas, the microtubules density, as measured by total α-tubulin immunofluorescence intensity in the M I spindles of Kif2a-knockdown oocytes was significantly higher compared to that in the control group (Fig. 8B), indicating that Kif2a destabilized the spindle microtubules in meiosis through microtubule depolymerization.

## Discussion

Kif2a is identified as a kinesin-13 microtubule depolymerase that regulates spindle organization, and chromosome movement in mitosis^17^,^27^. However, its requirements for oocyte meiotic maturation is poorly understood. Our work investigated the localization pattern and functions of Kif2a during mouse oocyte meiotic maturation. We showed that Kif2a was localized to the spindle microtubules, especially to the spindle poles and centromeres, and that it functions as major player of meiotic spindle assembly, chromosome alignment and segregation. Depletion of Kif2a by siRNA microinjection caused reduced microtubule depolymerization, disrupted meiotic spindles and chromosome misalignment, which in turn caused Pro-M I/M I stage arrest by activating SAC, as well as failure of first polar body extrusion.

In mitosis, Kif2a is localized to spindle microtubules and it is concentrated at spindle poles^11^, but at times to kinetochores^17^. We showed a similar distribution pattern of Kif2a in meiotic oocytes. We also detected Kif2a at the inner centromere from the first prometaphase I to the M II stage (Fig. 2). This localization pattern indicated that Kif2a may regulate spindle assembly and chromosome dynamics in mouse oocyte maturation.

To study the correlation between Kif2a and microtubule organization, we employed taxol and nocodazole. After taxol treatment, in addition to its localization to α-tubulin and abnormal spindle poles, Kif2a was also co-localized with cytoplasmic asters where it overlapped with γ-tubulin staining. This localization pattern was similar to that of the proteins involved in spindle formation, e.g. MDK^28^, PLK1^29^,^30^, Septin1^31^, BARC1^32^ and cep55^33^, all of which have been proved to participate in meiotic spindle assembly. When oocytes were exposed to nocodazole to disassemble microtubules, Kif2a staining partly dispersed into the cytoplasm and reappeared at the inner centromeres. These data suggested that Kif2a was strongly correlated with microtubule organization.

To determine the function of Kif2a in mouse oocyte meiosis, we used siRNA to knockdown Kif2a. Immunoblot analysis demonstrated that the Kif2a level was significantly reduced after siRNA injection. Knockdown of Kif2a resulted in severely disrupted spindles (e.g. nonpolar spindles, monopolar spindles, multipolar spindles and broadened spindles), and extensive chromosome misalignment. These results demonstrate that Kif2a plays an important role in spindle assembly and chromosome congregation. According to previous reports, Kif2a is a microtubule depolymerase, which disassembles microtubules at their minus ends at spindle poles, and this activity contributes to the poleward chromosome movement^27^. It was shown that Kif2a is essential for bipolar spindle assembly in mitosis, and perturbation of Kif2a led to monopolar spindle formation in mitosis^11^,^22^; it also at times led to multipolar spindles and failure in pole coalescence^17^. Meiotic systems have far more tubulin subunits than mitotic systems, and the depletion of Kif2a more likely results in disabled spindles in meiotic systems because excess microtubule polymerization overwhelms the meiotic machinery that organizes microtubules into the bipolar spindle^11^,^34^. Kif2a activity is dependent on its centrosome localization, which is regulated by the antagonistic regulations of Aurora A and Plk1, and it is also regulated through an interaction with inner centromere Kin-I stimulator (ICIS)^23^,^24^. Aurora B regulation of Kif2a was shown to control the length of midzone microtubules for ensuring faithful cell division^25^.

Spindle bipolarity is established and maintained by the concerted actions of motor and non-motor microtubule-associated proteins^35^. The maintenance of spindle bipolarity depends on the balance of opposing forces generated by Kif2a at spindle microtubule minus-ends at spindle poles and MCAK at plus-ends at kinetochores^16^,^36^. Loss of Kif2a leads to the absense of poleward force, and the remaining kinetochore-derived forces, resulting in abnormal meiotic spindles, such as monopolar spindles and nonpolar spindles. It could be inferred that Kif2a, through contributing to the dynamics of microtubules, has an important role in spindle assembly.

Another obvious phenotype of Kif2a depletion in oocyte includes multipolar spindles; cells with supernumerary centrosomes generate multipolar spindles. Robust mechanisms are in place that coalesce poles to assemble the multipolar spindles generated by extra centrosomes into bipolar spindles^37^. Recently, Kif2a was shown to have a role in centrosome clustering in Xenopus laevis embryos, where the depletion of Kif2a produced multipolar spindles^17^. Our data showed that Kif2a partly co-localizes with γ-tubulin at the abnormal spindle poles as well as in the cytoplasmic asters; after depletion of Kif2a (Fig. 3), γ-tubulin failed to cluster at spindle poles, but dispersed irregularly around the deformed spindles (Fig. 5).

Kif2a is required for stabilizing spindle microtubules. In vertebrate meiotic spindles, non-kinetochore microtubules (K-MT) comprise about 95% of the spindle microtubules. In order to investigate the role of Kif2a in the attachment of kinetochores to K-MT in meiosis, we performed cold treatment to depolymerize non-kinetochore microtubules^38^,^39^. Notably, a significant increase in microtubule density was observed in Kif2a-depleted MI oocytes after cold treatment (Fig. 8). The data indicates that Kif2a is critical for spindle dynamics through destabilizing microtubules, but not for kinetochore-MT attachments in meiosis. Our data strongly suggests that Kif2a may contribute to the assembly of a bipolar spindle through both microtubule dynamics and MTOC clustering.

We showed that perturbation of Kif2a also leads to a high frequency of misaligned chromosomes, and lagging chromosomes. The question is raised, why chromosome misalignment occurs? Spindle assembly defects may be implicated in the phenotypes, since the microtubule-based spindle structure is important for faithfull chromosome alignment and segregation in mitosis and meiosis^40^. Dynamic microtubule turnover of spindle microtubules is essential for congression of chromosomes at prometaphase, establishment of tension at metaphase, and segregation of chromosomes at anaphase^41^. Kif2a is involved in microtubule depolymerization at minus ends of the spindle to generate pole-migration forces for chromosome movement^21^,^25^.

Two important stages for the regulation of oocyte maturation to produce fertilizable eggs are prophase arrest release and progression through the M I stage^3^. The major components of SAC includes mitotic arrest-deficient-1 (Mad1), Mad2, budding-uninhibited by benzimidazole-1 (Bub1), Bub3, BubR1, monopolar spindle 1(Mps1)^42^,^43^. SAC contributes a surveillance mechanism to ensure proper chromosome segregation. The SAC pathway prevents chromosome missegregation by delaying the anaphase onset until all chromosomes are attached to spindle microtubules displaying proper tension and aligned at the equatorial plate^42^,^43^. Kif2a knockdown caused meiosis arrest at the Pro-M I/M I stage, and the PB1 failed to extrude. Furthermore, chromosome spreads confirmed that Kif2a knockdown leads to failure of homologous chromosome segregation. Bub3 signals were detected in the kinetochores of M I arrested oocytes in the Kif2a depletion group at 10 h of culture, while the signals were absent in the control group entering anaphase. Detection of Bub3 suggested that Pro-M I/M I arrest in the knockdown group was caused by the extended SAC activation due to abnormal spindles and significant chromosome misalignment.

In conclusion, our findings show that Kif2a, a kinesin-13 microtubule depolymerase, plays an important role in regulating spindle assembly, chromosome alignment and metaphase -anaphase transition in mouse oocyte meiotic maturation.

## Materials and Methods

### Antibodies and reagents

Rabbit polyclonal anti-Kif2a antibody used in Western blot and immunofluorescence was purchased from Novus Biologicals (Littleton,CO); Mouse monoclonal anti-α-tubulin-FITC antibody and mouse monoclonal anti-γ-tubulin antibody were obtained from Sigma-Aldrich Co. (Cat# F2168, Ca#T6557); Rabbit polyclonal anti-bub3 antibody was obtained from Santa Cruz Biotechology (Ca#sc-28258); Human polyclonal anti-centromere antibody (ACA) was obtained from Antibodies Incorporated (Item # 15-234-0001). Alexa Fluor@ 488-conjugate Goat anti-Rabbit IgG (H + L) and Alexa Fluor @594-conjugate Goat anti-Rabbit IgG (H + L) were produced by Thermo Fisher Scientific (Catalog# A-11008, Catalog# A-11012); Cy5-conjugated goat anti-human IgG and Cy5-conjugated goat anti-mouse IgG were purchased from Jackson ImmunoResearch Laboratory (West Grove, PA) (Cat#115-175-146, Cat#115-175-062). Mouse monoclonal anti-β-actin antibody was purchased from Santa Cruz Biotechnology (SC-8432, Santa Cruz, CA).

All other reagents were purchased from Sigma Aldrich except when mentioned otherwise.

### Oocyte collection and culture

The ovaries of female ICR mice (6–8 weeks old) were isolated, then cut with a blade. Only immature oocytes with intact germinal vesicles were collected in M2 medium with or without 100 μM IBMX. IBMX was used to maintain the GV oocytes at the GV stage. Then oocytes were cultured in M2 medium under liquid paraffin oil at 37 °C in an atmosphere of 5% CO_2_ in air for specific times.

### Micro-injection of Kif2a siRNAs

Microinjections were performed using a Narishige microinjector under a Nikon Diaphot ECLIPSE TE300 (Nikon UK Ltd.) and completed within 30 min. Small interfering RNAs (siRNAs) of Kif2a (Gene Pharma) were microinjected into the cytoplasm to knockdown Kif2a. The subsequent sequences of Kif2a siRNAs were used at 20 μM each, Kif2a siRNA-1: 5′-GGCCACUAGUGGAAACAAUTT-3′; Kif2a siRNA–2: 5′-GGAUGUUGAUGCUACAAAU TT-3′; Kif2a siRNA–3: 5′-GAAAACGACCACUCAAUAATT-3′. The same amount of negative control siRNAs was also injected as control. Then, oocytes were arrested at the GV stage in M2 medium supplemented with 100 μM IBMX for 24 h to knockdown Kif2a. Then the oocytes were thoroughly washed and transferred into IBMX-free M2 medium for further culture.

### Western blot analysis

A total of 200 mouse oocytes were collected in 5 μl 2 × SDS loading buffer and boiled for 5 min. Proteins were separated on SDS-PAGE and then transferred to PVDF (polyvinylidene difluoride) membranes, followed by washing briefly in TBST buffer. TBST containing 5% bovine serum albumin was used to block the membrane for 1 hour at room temperature. Then the membranes were incubated with 1:1000 rabbit polyclonal anti-Kif2a antibody, 1:2000 mouse monoclonal anti-β-actin antibody over night at 4 °C. After three washes in TBST buffer, 10 minutes each, the membranes were incubated with 1:1000 horseradish peroxidase (HRP)-conjugated goat anti-rabbit IgG at 37 °C for 2 h. Finally, the membranes were washed in TBST buffer for three times, and processed with the enhanced chemiluminescence (ECL) detection system (TGM) (Bio-RAD, CA).

### Taxol, nocodazole and cold treatment of oocytes

For taxol and nocodazole experiments, 5 mM taxol and 10 mg/ml nocodazole in DMSO stocks were diluted in M2 medium to achieve working concentrations. After 8 h or 12 h in culture, oocytes were treated with pre-warmed M2 medium containing nococazole (20 μg/ml) or taxol (10 μM). Incubation time for taxol treatment was 45 min and for nocodazole treatment was 10 min. Next, oocytes were washed thoroughly and fixed for immunofluorescent staining. The control group oocytes were treated with the same concentration of DMSO in the M2 medium.

For cold treatment, Kif2a siRNA injected and the control siRNA injected oocytes were arrested at the GV stage in M2 medium with 100 μM IBMX for 24 hours. After that, the oocytes were transferred to IBMX-free M2 medium for 8 hours of culture. Then the oocytes were incubated at 4 °C for 15 min or 30 min followed by immunofluorescent staining.

### Immunofluorescence and confocal microscopy

Oocytes were fixed in 4% paraformaldehyde in PBS with 0.5% Triton X-100 for 30 min at room temperature, followed by blocking in 1% BSA for 24 h at 4 °C. Thereafter oocytes were incubated with rabbit polyclonal anti-Kif2a antibody (Novus; 1:100), Mouse monoclonal anti-α-tubulin-FITC antibody (Sigma; 1:100), mouse monoclonal anti-γ-tubulin antibody (Sigma,1:100), rabbit polyclonal anti-bub3 antibody (Santa Cruz Biotechnology,1:50), human polyclonal anti-centromere antibody (ACA) (Antibodies Incorporated, 1:50), respectively, over night at 4 °C. After three washes (5 min each) in washing buffer (0.1% Tween 20 and 0.01% Triton X-100 in PBS), the oocytes were labeled with F594 conjugate goat anti-rabbit IgG (H + L) (Thermo Fisher Scientific,1:500), Cy5-conjugated goat anti-rabbit IgG (H + L) (Thermo Fisher Scientific,1:500), Cy5-conjugated goat anti-human IgG (Thermo Fisher Scientific, 1:500) for 2 h at room temperature, followed by three washes. Then oocytes were co-stained with Hoechst 33342 for 20 min.

For double staining of Kif2a/γ-tubulin and α-tubulin, after incubation with primary antibody of Kif2a/γ-tubulin, and three washes in washing buffer, oocytes were incubated with F594-conjugated goat anti-rabbit IgG/Cy5-conjugated goat anti-human IgG for 1 h at room temperature, and after three washes in washing buffer, the oocytes were blocked in 1% BSA for 1 h at room temperature, then stained with mouse monoclonal anti-α-tubulin-FITC antibody (Sigma; 1:100) for 2 h at room temperature. At last, the oocytes were co-stained with Hoechst 33342 for 20 min.

For double staining of Kif2a and ACA/γ-tubulin, after staining with Kif2a, oocytes were washed three times in washing buffer, then incubated with F594-conjugated goat anti-rabbit IgG (H + L) for 2 h at room temperature, and after three washes in washing buffer, oocytes were blocked again for 1 h at room temperature, then stained with primary antibody of ACA/γ-tubulin overnight at 4 °C. After washing three times, the oocytes were labeled with Cy5-conjugated goat anti-human IgG/Cy5-conjugated goat anti-mouse IgG for 2 h at room temperature. Finally, the oocytes were stained with Hoechst 33342 for 20 min after three washes in washing buffer.

For triple-staining of Kif2a, γ-tubulin and α-tubulin, the methods utilized were the same as described above. For staining of γ-tubulin and Kif2a, the oocytes were incubated with Cy5-conjugated goat anti-mouse IgG and F594 conjugated goat anti-rabbit IgG, respectively. DNA was stained with Hoechst 33342 for 20 min. Finally, oocytes were mounted on glass slides with anti-fade mounting medium (DABCO) which can retard photobleaching, and visualized with a confocal laser-scanning microscope (Zeiss LSM 780, Germany).

### Chromosome spreading

Oocytes were left in Tyrode’s solution (Sigma, T1788) at room temperature to remove the zona pellucida. After a brief recovery in M2 medium, the oocytes were dropped onto the slide and then fixed with a solution of 1% paraformaldehyde in distilled H_2_O (pH:9.2) containing 0.15% Triton X-100 and 3 mM dithiothreitol. The slides were dried slowly in a humid chamber, and blocked with 1% BSA for 1 h at room temperature. Thereafter, slides were incubated with rabbit polyclonal anti-Kif2a antibody (Novus;1:100), human polyclonal anti-centromere antibody (ACA) (Antibodies Incorporated, 1:50) overnight at 4 °C. After three washes in washing buffer, the slides were then incubated with the secondary antibodies (the second antibodies for Kif2a and ACA were F594-conjugated and Cy5-conjugated, respectively) for 2 h at room temperature. Hoechst 33342 was used for 20 min to stain chromosomes. The specimens were observed with a confocal laser-scanning microscope (Zeiss LSM 780, Germany).

### Statistical analysis

At least three replications were performed for each treatment. All percentages were expressed as means ± SEM and the number of oocytes observed (n) was put in parentheses. Data were analyzed by independent-sample *t*-test with SPSS 13.0 softwere (SPSS Inc.). P < 0.05 was considered to be statistically significant.

Fluorescence intensity statistics was conducted using ZEN (2012) software.

### Ethic statement

All procedures with mice were conducted in accordance with the Animal Research Committee of the Institute of Zoology, Chinese Academy of Sciences and approved by the Animal Ethics Committee of Chinese Academy of Sciences. All experimental protocols were conducted according to the Institute of Zoology, Chinese Academy of Sciences.

## Additional Information

**How to cite this article**: Yi, Z.-Y. *et al*. Kif2a regulates spindle organization and cell cycle progression in meiotic oocytes. *Sci. Rep.* **6**, 38574; doi: 10.1038/srep38574 (2016).

**Publisher's note:** Springer Nature remains neutral with regard to jurisdictional claims in published maps and institutional affiliations.

## Supplementary Material

Supplementary Information
