# Supplementary material for: Kif2a regulates spindle organization and cell cycle progression in meiotic oocytes
Source: Sci Rep. 2016 Dec 19;6:38574. doi: 10.1038/srep38574 (PMC5171826; doi:10.1038/srep38574)
Supplement: Supplementary Information [file srep38574-s1.pdf]

Kif2a regulates spindle organization and cell cycle progression in meiotic oocytes

Authors: Zi-Yun Yi[1], Xue-Shan Ma[2], Qiu-Xia Liang[2] ,Teng Zhang[2],Zhao-Yang Xu[2], Tie-Gang Meng[2],Ying-Chun Ouyang[2], Yi Hou[2], Heide Schatten[3], Qing-Yuan Sun[2] & Song Quan[1]

Author affliations:

[1]Center for Reproductive Medicine; Department of Ob/Gy; Nanfang Hospital; Southern Medical University; Guangzhou City, 510515 China;

[2]State Key Laboratory of Stem Cells and Reproductive Biology, Institute of Zoology, Chinese Academy of Sciences, Beijing, 100101 China;

[3]Department of Veterinary Pathobiology, University of Missouri, Columbia, MO 65211, USA

Correspondence and requests for materials should be addressed to S.Q. (email: quansong2008@gmail.com) or Q.Y.S. (email: sunqy@ioz.ac.cn).

Supplementary Information:

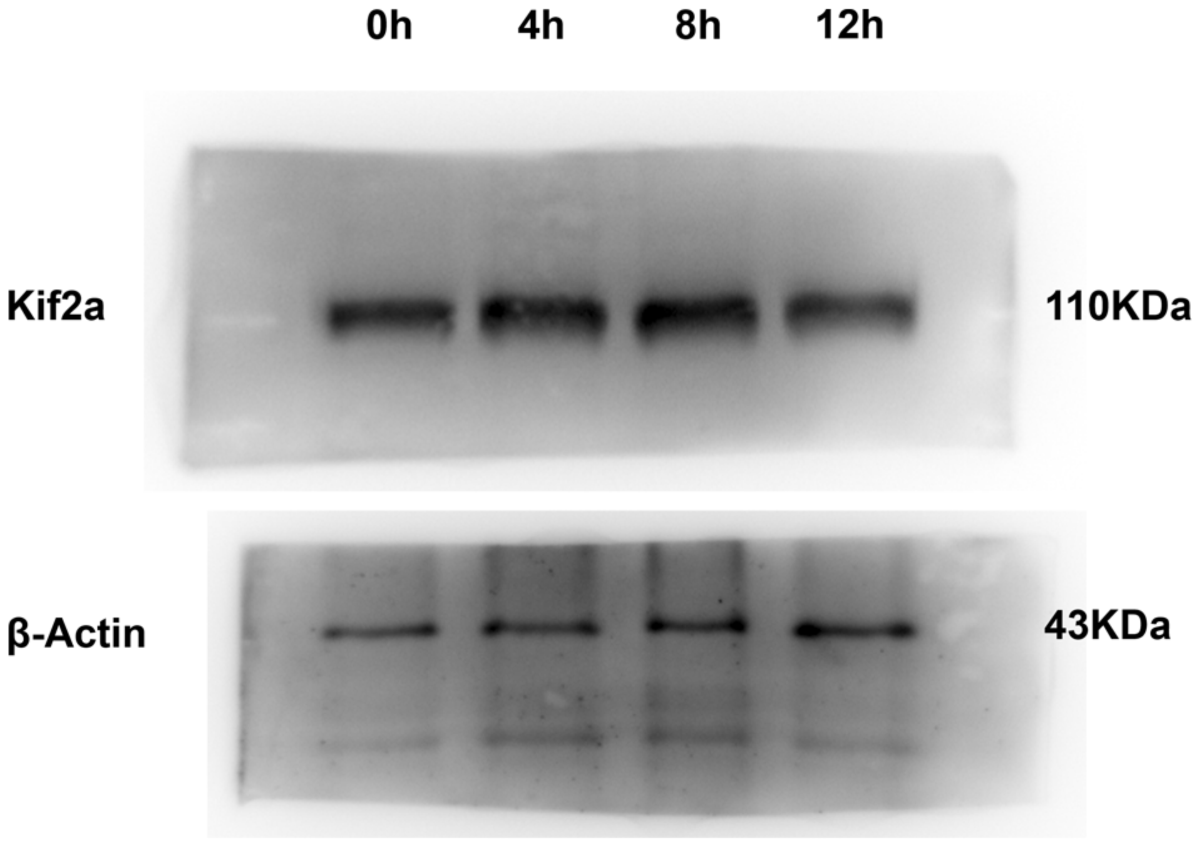

Supplementary Figure 1. Expression and subcellular localization of Kif2a during mouse oocyte meiotic maturation. Oocytes were collected after culture for 0, 4, 8, 12h, corresponding to the GV, Pro-MⅠ， MⅠand MⅡ stages, respectively. The molecular weight of Kif2a and β-actin were 110kDa and 43kDa, respectively. Each sample contained 200 oocytes.

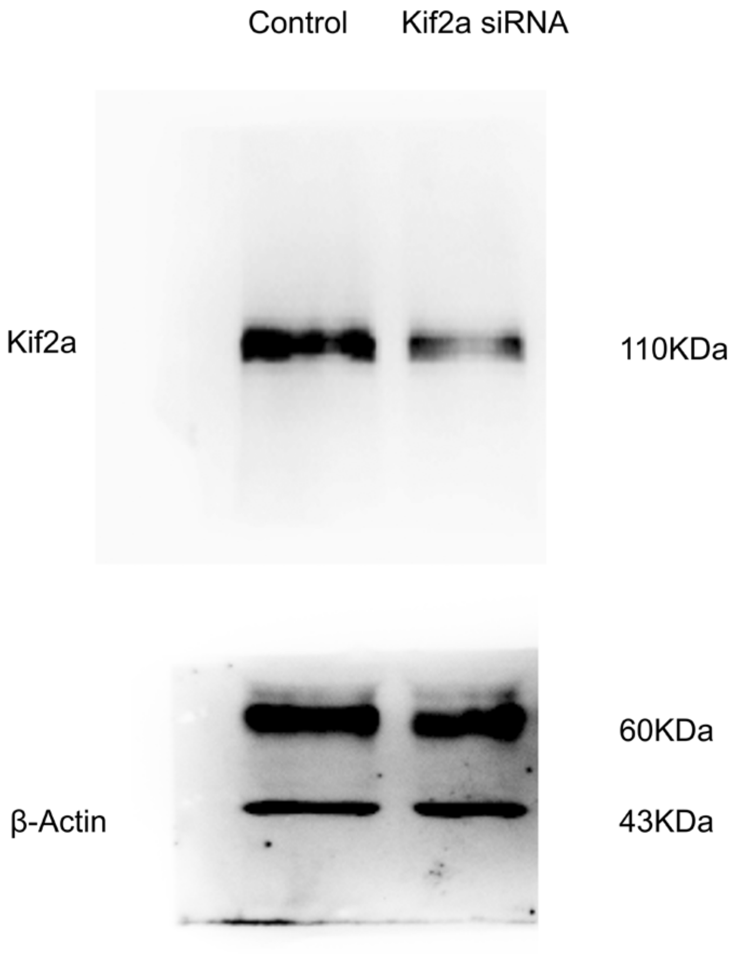

Supplementary Figure 2. Western blotting results for Kif2a after siRNA injection were Full-length gels. After microinjection of Kif2a or control siRNA , oocytes were incubated in M2 medium containing 100μm IBMX for 24h, then washed 5 times and placed in IBMX-free M2 medium for 8h, followed by Western blotting.
